# Glymphatic System Dysfunction Associated with Inflammatory Factors in Asymptomatic Neurosyphilis

**DOI:** 10.2174/0115734056454245260313071547

**Published:** 2026-03-30

**Authors:** Binbin Wang, Xiaoli Cui, Yuting Xia, Chunhua Xing, Shuo Li, Daixin Li, Chuanjun Xu, Chao Du, Yu-Chen Chen

**Affiliations:** 1 Department of Radiology, Nanjing First Hospital, Nanjing Medical University, Nanjing, China; 2 Department of Radiology, The Second Hospital of Nanjing, Nanjing University of Chinese Medicine, Nanjing, China; 3 Department of Neurology, Nanjing Yuhua Hospital, Yuhua Branch of Nanjing First Hospital, Nanjing, China

**Keywords:** Asymptomatic neurosyphilis, Inflammatory factors, Glymphatic dysfunction, DTI-ALPS, Normalized EPVS volume, Magnetic resonance imaging

## Abstract

**Introduction:**

This study seeks to delineate glymphatic system–related alterations in individuals with Asymptomatic Neurosyphilis (ANS) and to elucidate their correlation with inflammatory responses, with the objective of identifying early neuroimaging biomarkers indicative of neurosyphilis infection.

**Materials and Methods:**

In this prospective analysis, 33 patients with ANS and 29 matched healthy controls (HC) were enrolled from The Second Hospital of Nanjing. All participants underwent Magnetic Resonance Imaging (MRI) scanning. The diffusion tensor image analysis along the perivascular space (DTI-ALPS) indices and normalized enlarged perivascular spaces (EPVS) volume were quantified and compared between the ANS and HC groups. Furthermore, correlations were assessed between imaging-derived metrics and inflammatory profiles from both Cerebrospinal Fluid (CSF) and peripheral blood samples.

**Results:**

Marked differences in DTI-ALPS indices and normalized EPVS volume were evident between the ANS and HC groups. Post hoc analyses revealed that the ANS group exhibited significantly reduced DTI-ALPS indices (1.40 *vs* 1.60, *p* = 0.0001) and elevated normalized EPVS volume (2.28 *vs.* 2.05, *p* = 0.0355) relative to healthy individuals. In ANS, unadjusted analyses suggested that normalized EPVS volume demonstrated a positive correlation with CSF nucleated cell count (r = 0.413, *p* = 0.017), and a significant inverse relationship was observed between DTI-ALPS values and CSF lactate dehydrogenase (LDH) concentrations (r = -0.385, *p* = 0.027). However, after False Discovery Rate (FDR) correction, these correlations did not reach statistical significance.

**Discussion:**

The findings demonstrate, to our knowledge, for the first time, significant glymphatic system–related impairment in ANS. The reduced DTI-ALPS index suggests diminished glymphatic activity, while increased normalized EPVS volume points to structural perivascular alterations. The exploratory correlations between these imaging metrics and CSF inflammatory markers indicate a potential relationship between glymphatic system–related dysfunction and neuroinflammation in early neurosyphilis.

**Conclusion:**

Taken together, these findings indicate that distinct alterations in DTI-ALPS indices and normalized EPVS volume may represent novel neuroimaging biomarkers for the early detection and clinical evaluation of ANS. Utilization of these imaging indices could potentially enhance diagnostic precision and inform prognostic assessment in affected patients.

## 1 INTRODUCTION

Asymptomatic neurosyphilis (ANS) denotes a clinical state defined by CSF abnormalities, including heightened white blood cell counts, elevated protein concentrations, or positive syphilis serology, in the absence of overt neurological symptoms [1]. ANS may develop at any stage of syphilitic infection, particularly in individuals who have not received standard therapeutic interventions. Although patients lack clinical manifestations, accumulating evidence suggests that *Treponema pallidum* can invade the central nervous system (CNS) at an early stage, and delayed treatment may lead to irreversible sequelae. International guidelines therefore recommend routine CSF screening in high-risk individuals to enable early identification and intervention [2, 3].

The current diagnostic approach relies principally on serological assays and CSF analysis [1], which remain pivotal for detecting ANS. Nonetheless, these modalities are limited by suboptimal sensitivity and specificity, particularly during latent or incipient disease stages [4]. Moreover, lumbar puncture remains invasive, underscoring the need for non-invasive imaging markers capable of identifying early CNS involvement in ANS.

Several noninvasive MRI-derived indices enable indirect evaluation of glymphatic function, including enlarged perivascular spaces (EPVS), diffusion tensor image analysis along the perivascular space (DTI-ALPS), and choroid plexus volume (CPV). The glymphatic system-a brain-spanning perivascular network-mediates CSF influx along perivascular spaces (PVS), subsequent dispersion into the parenchyma, and efflux *via* perivenous routes, thus orchestrating solute clearance from the extracellular milieu [5, 6]. Anatomically, the glymphatic system incorporates PVS, which, when pathologically dilated, manifests as EPVS detectable by MRI. Quantification of EPVS by semi-automated or deep-learning models offers a robust structural marker of glymphatic system–related impairment. Clinical and epidemiological investigations have consistently associated higher normalized EPVS volume (particularly in the basal ganglia) with cognitive dysfunction, vascular risk profiles, and systemic or CNS inflammation [7, 8]. Nevertheless, recent population-level meta-analyses also underscore the complexity of this relationship, showing that the cognitive impact of EPVS may be small for total burden, but significant for region-specific or severe EPVS [9]. DTI-ALPS has emerged as a quantitative proxy for glymphatic activity, evaluating water diffusivity in various vectors within PVS, which is critical for normal CSF circulation [10]. PVS, comprising fluid-filled compartments enveloping cerebral microvessels, serve as conduits for fluid movement, enabling CSF–interstitial fluid exchange and metabolic waste elimination [11]. Inflammatory processes are recognized as central to the pathogenesis and exacerbation of PVS dysfunction [12]. Glymphatic system–related impairment is increasingly implicated in the pathophysiology of neurological disorders, including Parkinson’s disease and Alzheimer’s disease, among others [13-15].

The pathophysiological relationship between glymphatic dysfunction, neuroinflammation, and neural injury is increasingly evident. CNS infections trigger neuro-inflammation and can impair glymphatic function [16]. Recent studies show that reduced DTI-ALPS correlates with disease progression and is detectable preclinically, as in Creutzfeldt-Jakob disease [17], suggesting similar mechanisms may apply to ANS. However, dedicated research on ALPS and EPVS in this context is lacking. Given early, subclinical CNS infection and inflammation in neurosyphilis, subtle glymphatic system–related dysfunction detectable by these imaging markers may precede symptoms and predict progression. Therefore, further study of glymphatic imaging biomarkers in ANS could improve early detection, risk stratification, and mechanistic understanding of neuroinflammation-mediated glymphatic dysfunction To address this gap, this study sought, to our knowledge for the first time, to systematically assess alterations in the ALPS index and normalized EPVS volume, and to elucidate their associations with inflammatory biomarkers derived from both CSF and peripheral blood. This work seeks to clarify potential neural mechanisms underlying cognitive and sensory impairments in ANS. Such imaging abnormalities may represent early neuroimaging biomarkers of CNS injury in syphilis, thereby offering novel avenues for early diagnosis and disease monitoring.

## 2 MATERIALS AND METHODS

This investigation received ethical clearance from the Ethics Committee of The Second Hospital of Nanjing, and all participants or their legal representatives provided documented informed consent. The protocol adhered strictly to the guidelines articulated in the Declaration of Helsinki.

### 2.1 Participants

Thirty-three individuals diagnosed with ANS were prospectively enrolled from The Second Hospital of Nanjing between January 2023 and February 2024. An additional 29 healthy controls (HC) were identified through targeted community health campaigns and advertisement initiatives, selected to closely match patient demographics in terms of age, sex, educational attainment, and handedness. The sample size for this initial investigation was determined primarily based on feasibility and logistical considerations over the study period, and is comparable to those reported in previous pioneering neuroimaging studies of glymphatic function. None of the subjects exhibited neurological deficits, and all performed within the normative range on cognitive screening, corroborated by Mini-Mental State Examination (MMSE, score ≥24) and Montreal Cognitive Assessment (MoCA, score ≥26). The study followed the Sex and Gender Equity in Research guidelines (SAGER) to ensure appropriate inclusion and reporting of sex-related data.

Eligibility requirements included: high-risk sexual behaviors, multiple sexual partners, sexually transmitted disease history, or receipt of blood transfusions; the absence of clinical neurological manifestations; and laboratory evidence comprising: (1) positive non-treponemal serology (typically present, except in rare advanced-stage cases), (2) positive treponemal serology, (3) CSF anomalies such as leukocyte count ≥5×10^6^/L or protein concentration >500 mg/L, excluding other etiologies; positive *treponema pallidum* particle agglutination assay (TPPA) and toluidine red unheated serum test (TRUST) outcomes; age 20 to 80 years; and an educational background spanning 4 to 20 years.

Exclusion criteria encompassed: Conventional magnetic resonance imaging shows significant brain atrophy or cerebral infarction; prior diagnoses of psychiatric disorders, substance dependence, hepatic or endocrine abnormalities, or additional intracranial pathologies; conditions known to yield false-positive syphilitic serologies (*e.g.*, acute febrile episodes, connective tissue disorders); and contraindications to MRI, including pacemaker implantation, metallic hardware, or claustrophobic tendencies.

### 2.2 Neuropsychological Evaluation

All subjects completed a standardized neuropsychological protocol administered in a distraction-minimized environment, utilizing validated Chinese versions of the MMSE and MoCA. Assessments were conducted in a consistent sequence by trained personnel to ensure methodological reliability.

### 2.3 Peripheral Blood and CSF Sampling with Inflammatory Marker Analysis

Fasting peripheral venous blood was drawn into two-potassium EDTA tubes and processed at 4000 rpm for five minutes to separate plasma, which was then aliquoted and preserved at -80°C. Hematological indices, including white blood cell, neutrophil, and lymphocyte counts, were quantified through an automated system. Derived indices, such as neutrophil-lymphocyte ratio (NLR) and platelet-lymphocyte ratio (PLR), were subsequently computed.

For CSF acquisition, lumbar puncture was performed at the L3–4 or L4–5 interspaces in subjects with ANS, with 1–2 ml obtained per sample tube. Stringent handling procedures were implemented to curtail cellular degradation or erythrocyte admixture, and all specimens were submitted for laboratory analysis within sixty minutes of collection.

### 2.4 MRI Data Acquisition

Neuroimaging was performed on a 3.0T Signa Pioneer MRI system equipped with a 24-channel phased-array head coil. High-resolution 3D-BRAVO T1-weighted images were captured employing the following sequence parameters: repetition time (TR) 8.6 ms, echo time (TE) 3.3 ms, slice thickness 1.8 mm, field of view (FOV) 240 × 240 mm^2^, matrix size 256 × 224, and flip angle (FA) 12°.

For DTI, a two-dimensional spin-echo echo-planar imaging (SE-EPI) protocol was utilized, TR 4300 ms, TE 79 ms, b values of 0 and 1000 s/mm^2^, FOV 240 × 240 mm^2^, matrix 128 × 128, slice thickness 4 mm, 35 contiguous slices, and 30 unique diffusion-encoding directions.

### 2.5 Imaging Analysis

#### 2.5.1 DTI-ALPS Index Computation

**Fig. (1):**
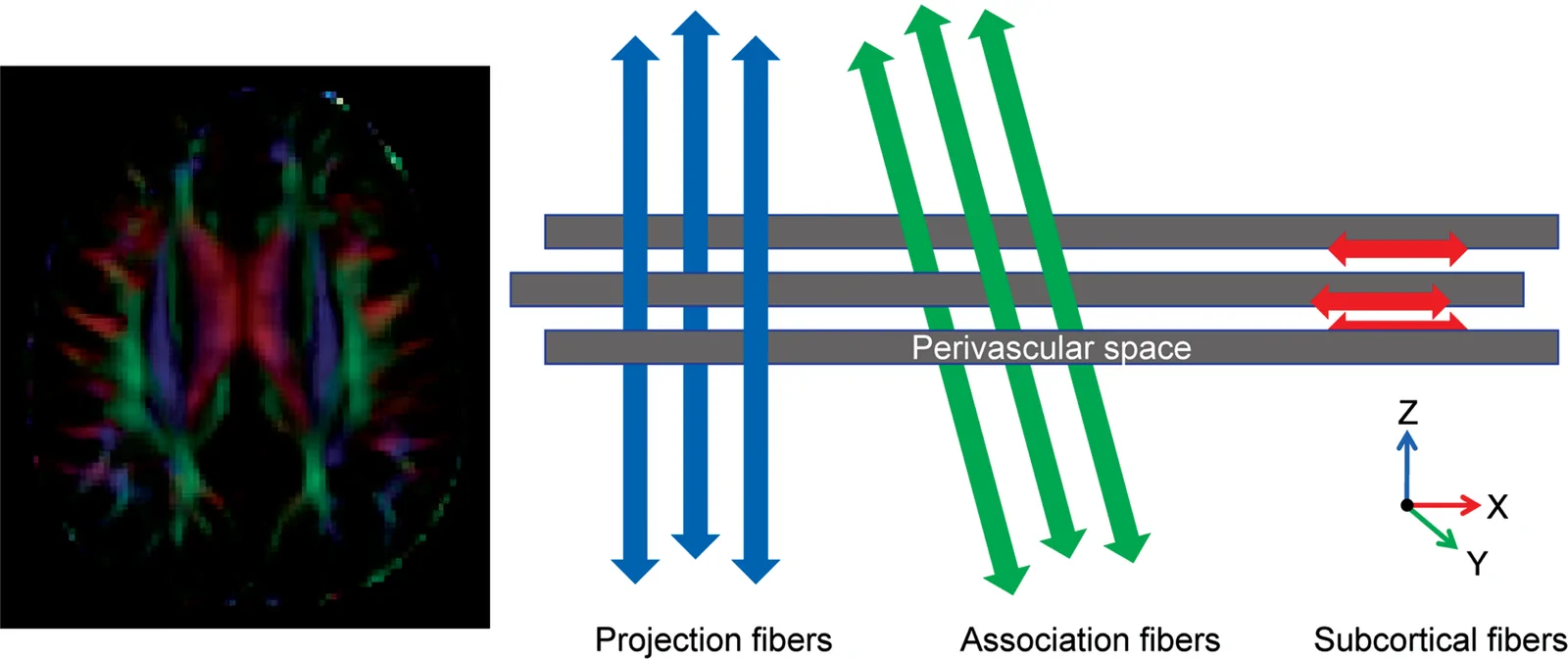
DTI-ALPS calculation method.

Data were processed using FSL version 6.0.3 (http://www.fmrib.ox.ac.uk/fsl). Preprocessing steps included b0 extraction, brain extraction, head motion, and eddy-current artifact correction, followed by tensor modeling *via* the ‘dtifit’ tool, yielding directionally specific diffusion coefficient maps (Dxx, Dyy, Dzz). Diffusion datasets were initially coregistered to individual high-resolution 3D T1-weighted images using FMRIB's Linear Image Registration Tool (FLIRT) and subsequently normalized to standard space *via* nonlinear deformation (FNIRT). Standard-to-native image transformations were performed with inverse warp fields and affine matrices. Figure **1** shows the process for obtaining the DTI-ALPS index.

ALPS indices were quantified within native DTI space. Four spherical regions of interest (ROIs), each 5 mm in diameter, representing bilateral association and projection fiber bundles, were identified based on coordinates from the JHU ICBM FA template and spatially registered [18]. An experienced radiologist (over a decade of diagnostic imaging expertise) verified all registrations; manual adjustments were executed as necessary. Calculation of the ALPS index was conducted using the equation:

ALPS = mean (Dxx_projection, Dxx_association) / mean (Dyy_projection, Dzz_association),

with final values derived from bilateral averaging.

### 2.6 Quantification of EPVS

Automated EPVS segmentation was performed using a validated deep learning algorithm developed by Boutinaud *et al*. [19], operating directly on three-dimensional T1-weighted images. Preprocessing included FreeSurfer(v6.0) tissue segmentation to obtain gray matter, white matter, CSF, and intracranial volume (ICV), followed by 0–1 intensity normalization using the 99th percentile. Non-brain structures were removed *via* an automatically generated bounding box, and basal ganglia (BG) masks were derived from FreeSurfer labels for regional classification. EPVS segmentation used a 3D U-Net architecture pretrained with a convolutional autoencoder. The autoencoder was trained on 1,782 unlabeled T1 scans, and supervised U-Net training employed 40 manually annotated scans with five-fold cross-validation. Final EPVS probability maps were generated by averaging outputs from all folds to obtain a consensus prediction. Probability maps were binarized using a validated threshold of 0.1, and EPVS clusters were extracted using 26-connected neighborhood criteria. EPVS total volume, cluster count, and normalized EPVS volume (EPVS volume/ICV) were computed. EPVS volume was additionally quantified separately within deep white matter (DWM) and BG regions.

### 2.7 Quantification of Choroid Plexus Volume (CPV)

CPV was quantified as an indicator of CSF production and blood-CSF barrier immune signaling activity. To minimize errors introduced by manual segmentation, we applied an automated segmentation method based on a Convolutional Neural Network (CNN) employing a U-Net architecture, specifically designed to identify the choroid plexus in T1-weighted (T1w) MRI scans (code available at: https://github.com/Center-of-Imaging-Biomarker-Development/chp_seg) [20]. This neural network framework has been previously validated in adult populations across different ages and has demonstrated accuracy superior to traditional segmentation algorithms. Briefly, each subject's T1-weighted image was first non-linearly registered to the ICBM-MNI 152 T1-weighted brain template using the Advanced Normalization Tools (ANTs) software suite [21]. The registered images were then input into the 3D U-Net network to generate choroid plexus maps, which were subsequently inverse-transformed back to the original anatomical space. This transformation generated individualized choroid plexus masks. The resulting masks were visually inspected and validated by a trained imaging analyst. Finally, the final volume was calculated in Python (version 3.7.0) using the SimpleITK package (version 2.1.1.1), yielding the CPV for each participant [22]. To account for overall inter-individual differences in brain size, we measured Total Intracranial Volume (TIV), Gray Matter Volume (GMV), White Matter Volume (WMV), and CSF Volume (CSFV) using the Computational Anatomy Toolbox (CAT12) within the Statistical Parametric Mapping framework (SPM12). This toolbox, developed by the Department of Psychiatry and Neurology at Jena University Hospital, Germany, provides a robust platform for voxel-based morphometry. All measurements were normalized by expressing the volume as a percentage of TIV multiplied by 1000, thereby controlling for inter-individual variations in brain size. Subsequent analyses were based on these normalized metrics.

### 2.8 Statistical Analysis

Analysis was conducted using SPSS v25.0 (IBM, Armonk, NY, USA) and GraphPad Prism v10.2.0 (GraphPad Software, Boston, MA, USA; www.graphpad.com). The statistical significance level was established at a two-tailed *p* < 0.05.

Normal distribution of continuous variables was verified utilizing the Shapiro-Wilk test. Student’s t tests were performed to compare the differences in normally distributed continuous data, and Mann-Whitney U tests were used to compare non-normally distributed data. The chi-square test was performed to test for differences in categorical metrics. To explore associations among peripheral inflammatory parameters, CSF markers, DTI-ALPS indices, Normalized EPVS volumes, and clinical characteristics in ANS, Spearman’s rank correlation coefficients were calculated. To control for type I error in the correlation analyses, multiple-comparison adjustment was performed using the False Discovery Rate (FDR) correction; adjusted *p*-values (q-values) < 0.05 were considered statistically significant.

## 3 RESULTS

### 3.1 Demographic and Clinical Characteristics

**Fig. (2):**
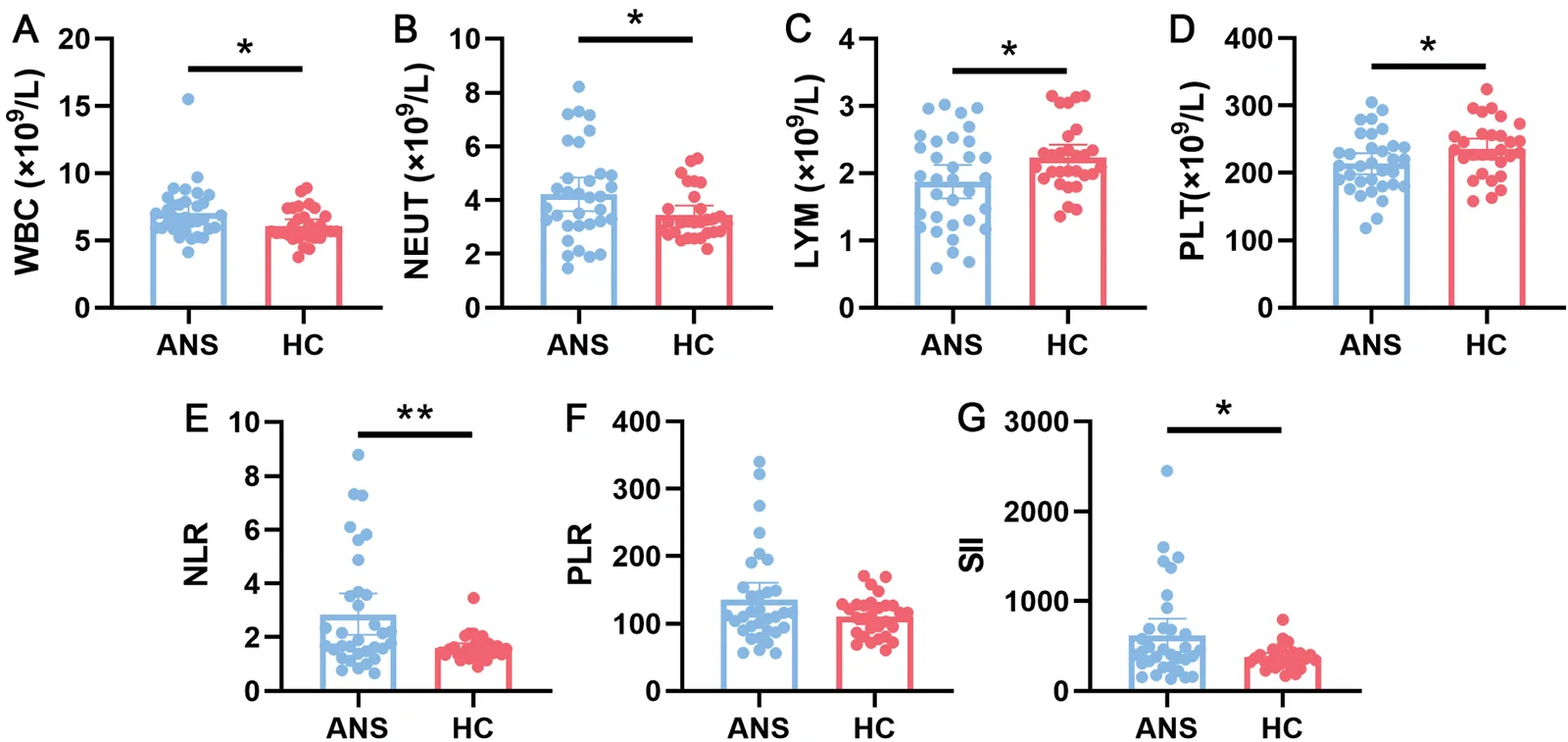
Comparison of hematological and inflammatory indices between ANS and HC. Data are presented as scatter plots with mean ± 95% confidence interval (CI). Each dot represents an individual subject (ANS, n = 33; HC, n = 29). (**A**) White blood cell count (WBC, ×10^9^/L). (**B**) Neutrophil count (NEUT, ×10^9^/L). (**C**) Lymphocyte count (Lym, ×10^9^/L). (**D**) Platelet count (PLT, ×10^9^/L). (**E**) Neutrophil-to-lymphocyte ratio (NLR). (**F**) Platelet-to-lymphocyte ratio (PLR). (**G**) Systemic immune-inflammation index (SII). * *p* < 0.05, ** *p* < 0.01.

This study included 33 patients with ANS and 29 healthy control participants (Table **1**). There were no significant differences between the ANS and HC groups in age, gender distribution, or years of education, indicating comparable baseline characteristics. Peripheral blood analysis revealed that, relative to healthy controls, ANS had significantly increased white blood cell count (7.06 ± 1.95 *vs.* 6.10 ± 1.21 ×10^9^/L, *p* = 0.027), neutrophil count (4.21 ± 1.72 *vs.* 3.44 ± 0.91 ×10^9^/L, *p* = 0.037), and systemic immune-inflammation index (SII, 618.68 ± 514.85 *vs.* 374.37 ± 125.16, *p* = 0.017), while lymphocyte (1.87 ± 0.69 *vs.* 2.23 ± 0.49 ×10^9^/L, *p* = 0.026) and platelet (213.73 ± 43.57 *vs.* 235.62 ± 40.45 ×10^9^/L, *p* = 0.049) counts were notably decreased. Furthermore, the NLR was substantially higher in ANS (2.85 ± 2.13 *vs.* 1.59 ± 0.47, *p* = 0.003), highlighting a pronounced inflammatory state. Although the PLR was elevated in neurosyphilis patients (135.28 ± 70.24 *vs.* 110.40 ± 29.08), this difference did not reach statistical significance (*p* = 0.085). Cerebrospinal fluid parameters, available only for neurosyphilis patients, reflected elevated total protein (536.61 ± 179.40 mg/L), nucleated cell count (12.97 ± 13.59 ×10^6^/L), LDH (19.45 ± 6.01 IU/L), adenosine deaminase (ADA) (1.15 ± 1.52 U/L), and serum TRUST ratio (21.61 ± 20.36) (Fig. **2**), supporting central nervous system immune activation in this cohort.

### 3.2 Intergroup Differences in MRI Measurements

**Fig. (3):**
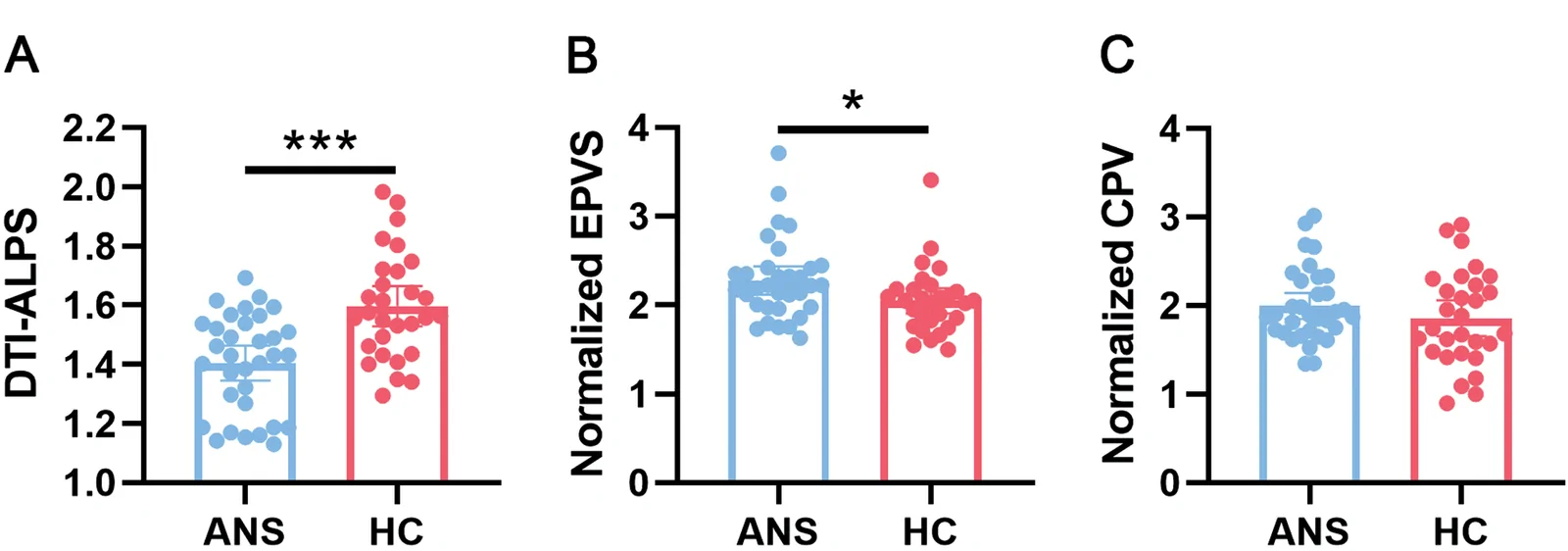
Comparison of neuroimaging lymphatic markers between HC and ANS groups. Data are presented as scatter plots with mean ± 95% CI. (**A**) DTI-ALPS index in HC and ANS. (**B**) Normalized EPVS volume in HC and ANS. (**C**) Normalized CPV in HC and ANS. * *p* < 0.05, *** *p* < 0.001.

Compared with healthy controls, ANS exhibited significant alterations in neuroimaging-derived glymphatic system–related metrics. Although the normalized CPV was slightly higher in ANS (2.00 ± 0.42) compared to HC (1.86 ± 0.53), this difference was not statistically significant (*p* = 0.2577). In contrast, the DTI-ALPS index, which reflects glymphatic system activity, was significantly reduced in ANS (1.40 ± 0.17) relative to HC (1.60 ± 0.18, *p* = 0.0001), indicating impaired glymphatic pathway function. Additionally, ANS showed significantly increased normalized EPVS volume (2.28 ± 0.45 *vs.* 2.05 ± 0.38, *p* = 0.0355), highlighting structural changes consistent with altered perivascular clearance (Table **2**, Fig. **3**).

### 3.3 Correlation Analysis

**Fig. (4):**
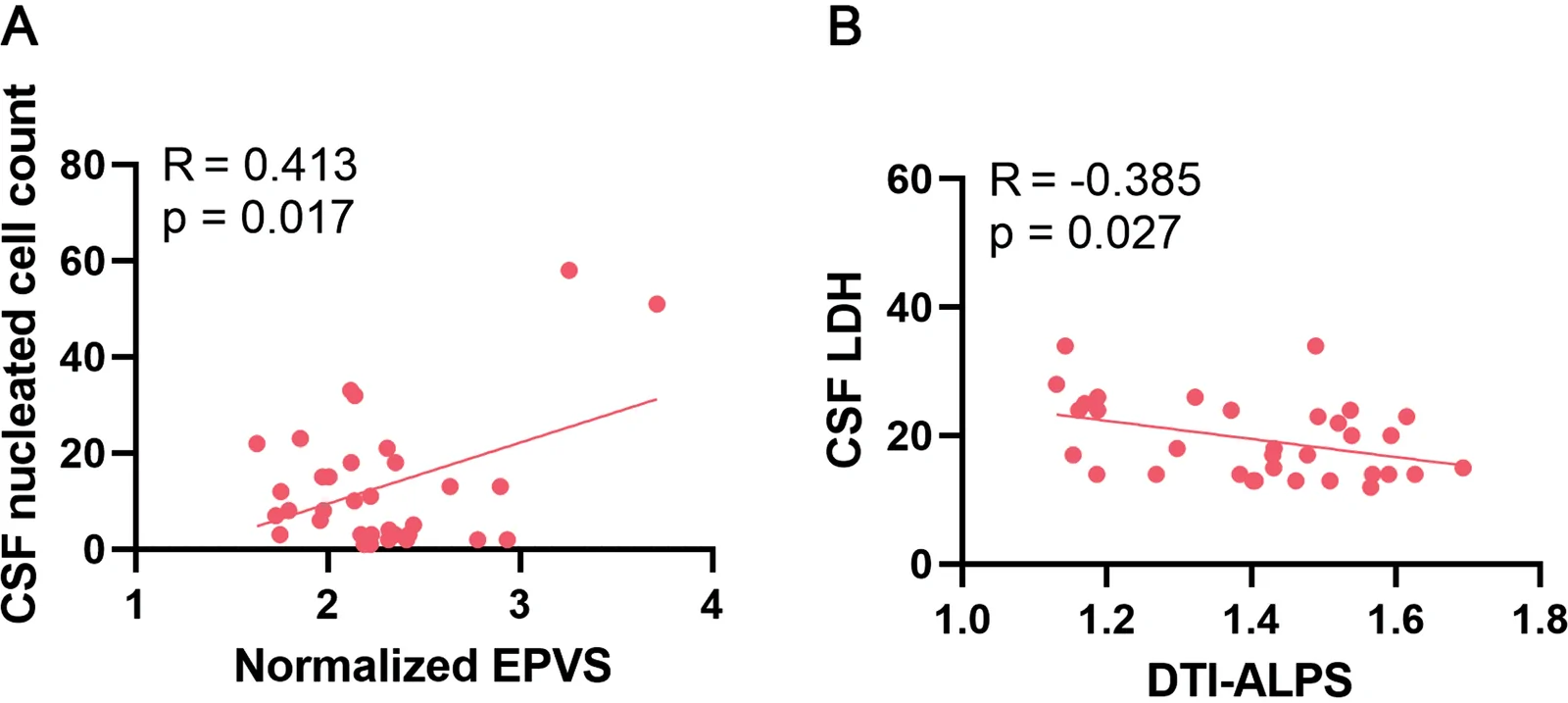
Correlations between neuroimaging lymphatic indices and CSF inflammatory markers in ANS. (**A**) Normalized EPVS volume versus CSF nucleated cell count. (**B**) DTI-ALPS versus CSF LDH. Each point represents an individual subject. Correlation coefficients (R) and *p*-values are shown (unadjusted).

Correlation analyses revealed distinct relationships between glymphatic imaging indices and CSF inflammatory markers in ANS. In unadjusted analyses, higher normalized EPVS volume was positively correlated with increased CSF nucleated cell count (r = 0.413, *p* = 0.017). In contrast, a lower DTI-ALPS index was associated with higher CSF LDH levels (r = -0.385, *p* = 0.027) (Fig. **4**). However, after applying FDR correction for multiple comparisons (correcting for 33 comparisons), these correlations did not remain statistically significant (all q-values > 0.05). Consequently, these associations should be considered exploratory and interpreted with caution.

## 4 DISCUSSION

The present study provides novel insights into the neuroimaging characteristics of ANS, highlighting significant alterations in DTI-ALPS indices and normalized EPVS volume relative to HC. These findings are of particular importance, as the early identification of CNS involvement in neurosyphilis has long posed clinical challenges, often resulting in delayed diagnosis and suboptimal patient outcomes [4]. The main finding of this study is, to our knowledge, the first demonstration that neurosyphilis is associated with significant impairment of glymphatic system–related indices. Dysfunction of the glymphatic system can be detected even in the early, asymptomatic stage of infection.

Previous research has demonstrated that glymphatic dysfunction may contribute to aberrant clearance of metabolites and immune mediators in neurological disease, potentially exacerbating neural injury and cognitive decline [11]. The DTI-ALPS index offers a quantitative approach for assessing glymphatic system function and has been applied to investigate glymphatic alterations in conditions including Alzheimer’s disease, Parkinson’s disease, hearing loss, and malignancies [10, 23-26]. Comparably, the observed reduction in DTI-ALPS indices among ANS suggests early impairment in glymphatic activity. In addition, attenuation of DTI-ALPS indices observed in ANS is potentially attributable to low-grade meningeal inflammation induced by *Treponema pallidum*, altered CSF dynamics, or inflammation-associated parenchymal edema, each contributing to disrupted fluid homeostasis [5, 6].

It is crucial to distinguish the pathophysiological implications of these two markers, as they likely reflect different aspects of glymphatic dysfunction in neurosyphilis. DTI-ALPS primarily captures the functional diffusivity of water along perivascular channels, representing the dynamic efficiency of fluid flow. In contrast, EPVS represents a structural accumulation of fluid and perivascular space dilation. In the context of ANS, acute or subacute inflammation triggered by *Treponema pallidum* invasion may initially disrupt the kinetic properties of the glymphatic system (lowering the ALPS index) due to cellular infiltration or micro-edema. Conversely, the structural enlargement of PVS (increased normalized EPVS volume) may result from chronic accumulation of inflammatory debris or long-standing impairment of blood-brain barrier integrity [27, 28]. Thus, while both markers are interrelated, they offer complementary insights into the structural-functional uncoupling of the glymphatic system in early infection.

EPVS have been associated with blood-brain barrier disruption and are increasingly considered a marker of microvascular pathology and CNS inflammation [29]. Increased normalized EPVS volume is indicative of impaired perivascular drainage, aberrant CSF kinetics, or the presence of interstitial swelling. Despite an absence of overt neurological deficits in ANS, the heightened normalized EPVS volume signals subclinical inflammation and incipient obstruction within CNS clearance systems. Indeed, *T.pallidum*–driven perturbations frequently undermine meningeal architecture and compromise blood–brain barrier integrity, facilitating cellular ingress into perivascular compartments and, in turn, EPVS dilation [27, 28]. These imaging phenomena affirm the presence of subtle neuropathological alterations in early neurosyphilis, detectable prior to clinical manifestation [12, 30].

CSF analysis is a valuable tool for diagnosing various neurological diseases. An increased CSF nucleated cell count may reflect a range of central nervous system pathologies, such as infectious processes and inflammatory responses [31, 32]. Moreover, CSF LDH is frequently employed as a diagnostic marker for various central nervous system disorders. Increased CSF LDH concentrations often signal neuronal or glial injury resulting from infection, inflammatory processes, or neoplastic activity [33-35]. A direct association between normalized EPVS volume and CSF nucleated cell counts, along with an inverse relationship between DTI-ALPS values and CSF LDH concentrations observed in the unadjusted analysis, demonstrates that glymphatic imaging markers may serve as potential indicators of early neuroinflammation and neuropathological changes induced by neurosyphilis.

From a clinical perspective, early recognition and intervention remain central to effective neurosyphilis management. Glymphatic system impairment has previously been implicated in a range of neurological disorders and cognitive deterioration, such as Parkinson's disease and stroke [36-39]. Notably, our study is the first to demonstrate that glymphatic system-related dysfunction is already present in neurosyphilis during its asymptomatic phase, indicating that cerebral glymphatic evaluation may offer potential sensitivity for detecting early-stage neurosyphilis. Regarding clinical translation, it is important to address whether these imaging biomarkers could replace lumbar puncture (LP). Given that LP remains the gold standard for confirming *Treponema pallidum* invasion and assessing specific serological markers, these imaging indices should currently be viewed as complementary tools rather than replacements. However, in selected clinical scenarios-such as monitoring treatment response or in patients for whom repeat LP is contraindicated or refused-non-invasive glymphatic imaging could provide a valuable surrogate marker to stratify risk and assess the severity of neuroinflammation.

## 5 LIMITATIONS

Nonetheless, several limitations should be acknowledged. First, the relatively small sample size and single-center design limit generalizability, and differences in imaging protocols or analysis pipelines may affect reproducibility. Specific power calculations were not performed a priori due to the exploratory nature of the study, which may limit the statistical power to detect smaller effects. Second, although DTI-ALPS indices and normalized EPVS volume are promising, they are influenced by multiple biologic variables (such as age, vascular risk factors including hypertension and diabetes, smoking status, and sleep quality). Due to the limited sample size, we were unable to fully adjust for these potential confounders in multivariate analyses; future studies with larger cohorts are needed to validate these findings. Third, the mechanisms underlying the observed glymphatic dysfunction in neurosyphilis remain speculative; while neuroinflammation is a candidate, other factors such as perivascular fibrosis, direct *Treponema pallidum* invasion, or astrocytic dysfunction warrant mechanistic investigation [40-42]. Fourth, longitudinal studies are needed to determine whether these imaging alterations predict progression to symptomatic neurosyphilis or respond to antimicrobial and anti-inflammatory therapy, thereby validating their prognostic value [43]. Fifth, regarding statistical analysis, although we observed correlations between imaging and inflammatory markers in unadjusted tests, these did not survive strict FDR correction for multiple comparisons. This suggests that the reported associations should be viewed as exploratory and require replication in independent samples. Lastly, integrating neuroimaging biomarkers with other emerging tools-such as neurofilament light chain or tau biomarkers in CSF, advanced connectomic analyses, and machine learning classifiers-could further enhance early diagnosis and therapeutic guidance in neurosyphilis and other infectious or inflammatory brain diseases.

## CONCLUSION

In summary, our findings indicate that the DTI-ALPS index and normalized EPVS volume are altered in patients with asymptomatic neurosyphilis and may correlate with CSF inflammatory markers. These non-invasive imaging metrics show potential as supplementary biomarkers for identifying early central nervous system involvement in neurosyphilis, warranting further validation in larger cohorts.

## Figures and Tables

**Table 1:** Demographic, clinical, and hematological characteristics of ANS and HC.

Items	ANS(n=33)	HC(n=29)	*p*-value
Age (year)	41.52 ± 15.23	39.90 ± 13.71	0.66
Gender (male: female)	30/3	26/3	0.87
Education (years)	13.64 ± 4.09	14.83 ± 4.61	0.29
MMSE	27.03 ± 0.97	26.79 ± 1.00	0.35
MOCA	27.64 ± 0.98	27.76 ± 0.93	0.62
CSF total protein	536.61 ± 179.40	-	-
Serum TRUST Ratio	21.61 ± 20.36	-	-
CSF nucleated cell count	12.97 ± 13.59	-	-
CSF LDH	19.45 ± 6.01	-	-
CSF ADA	1.15 ± 1.52	-	-
CSF Cl^−^	123.95 ± 3.19	-	-
CSF Glucose	3.45 ± 0.47	-	-
WBC (×10^9^/L)	7.06 ± 1.95	6.10 ± 1.21	0.027*
NEUT (×10^9^/L)	4.21 ± 1.72	3.44 ± 0.91	0.037*
Lym (×10^9^/L)	1.87 ± 0.69	2.23 ± 0.49	0.026*
PLT(×10^9^/L)	213.73 ± 43.57	235.62 ± 40.45	0.049*
NLR	2.85 ± 2.13	1.59 ± 0.47	0.003**
PLR	135.28 ± 70.24	110.40 ± 29.08	0.085
SII	618.68 ± 514.85	374.37 ± 125.16	0.017*

**Note:** Values are presented as mean ± standard deviation. Statistical significance is indicated by * *p* < 0.05 or ** *p* < 0.01. Comparisons were performed using Student’s t-test for continuous variables (Age, Education, and blood indices) and Chi-square test for categorical variables (Gender). ANS, asymptomatic neurosyphilis; HC, healthy controls; CSF, cerebrospinal fluid; LDH, lactate dehydrogenase; ADA, adenosine deaminase; WBC, white blood cell count; NEUT, neutrophil count; Lym, lymphocyte count; PLT, platelet count; NLR, neutrophil-to-lymphocyte ratio; PLR, platelet-to-lymphocyte ratio; SII, systemic immune-inflammation index. Note: CSF parameters were assessed only in the ANS group. NLR=NEUT /Lym; PLR = PLT / Lym; SII = PLT x NEUT / Lym.

**Table 2:** Comparisons of neuroimaging-derived glymphatic system–related metrics between ANS and HC groups.

Items	ANS(n=33)	HC(n=29)	*p*-value
Normalized CPV	2.00 ± 0.42	1.86 ± 0.53	0.2577
DTI-ALPS	1.40 ± 0.17	1.60 ± 0.18	0.0001***
Normalized EPVS	2.28 ± 0.45	2.05 ± 0.38	0.0355*

**Note:** Values are presented as mean ± standard deviation. Statistical significance is indicated by * *p* < 0.05 or *** *p* < 0.001. Comparisons were performed using Student’s t-test. ANS, asymptomatic neurosyphilis; HC, healthy controls; CPV, choroid plexus volume; DTI-ALPS, diffusion tensor image analysis along the perivascular space; EPVS, enlarged perivascular spaces. Normalized EPVS volume represents the total EPVS volume normalized to intracranial volume.

## Data Availability

The data that support the findings of this study are available from the corresponding authors, [Y.C.C] and [C.D] on special request.

## LIST OF ABBREVIATIONS

ANS: Asymptomatic neurosyphilis
HC: Healthy controls
MRI: Magnetic resonance imaging
DTI-ALPS: Diffusion tensor image analysis along the perivascular space
EPVS: Enlarged perivascular spaces
CPV: Choroid plexus volume
CSF: Cerebrospinal fluid
CNS: Central nervous system
MMSE: Mini-Mental State Examination
MoCA: Montreal Cognitive Assessment
NLR: Neutrophil-lymphocyte ratio
PLR: Platelet-lymphocyte ratio
TR: Repetition time
TE: Echo time
FOV: Field of view
FA: Flip angle
LDH: Lactate dehydrogenase
TPPA: *Treponema pallidum* particle agglutination assay
TRUST: Toluidine red unheated serum test
ADA: Adenosine deaminase

## STANDARDS OF REPORTING

STROBE guidelines were followed.
